# RETRACTION: Anti‐Inflammatory and Healing Activity of the Hydroalcoholic Fruit Extract of Solanum diploconos (Mart.) Bohs

**DOI:** 10.1155/jimr/9801373

**Published:** 2026-02-06

**Authors:** Journal of Immunology Research

RETRACTION: L. Benvenutti, R. Nunes, I. Venturi, et al., “Anti‐Inflammatory and Healing Activity of the Hydroalcoholic Fruit Extract of Solanum diploconos (Mart.) Bohs,” *Journal of Immunology Research*, 2021 (2021). https://doi.org/10.1155/2021/9957451.

The above article, published online on 20 July 2021 in Wiley Online Library (wileyonlinelibrary.com), has been retracted following the agreement of the journal’s Chief Editor Nadja Bakocevic; and John Wiley & Sons Ltd.

The retraction has been agreed following an investigation of the concerns raised by *Archasia belfragei* on PubPeer [1], which identified concerns related to Figure 4. More specifically, the histological samples shown in panels d–i appear identical to the tissue in Figure 7d–i of [2], in which they depict a different magnification. Further investigation has additionally identified unexpected similarities between the 1 µg/mL and 10 µg/mL S.d. panels at time 0 in Figure 5a.

Following our request to provide the raw data supporting the affected figures, the authors were unable to do so. The authors have since repeated the experiments described in the paper on their own initiative and provided the corresponding data, however this was not sufficient to address our concerns.

The authors disagree with this retraction.
